# Lectures on Natural and Difficult Parturition

**Published:** 1846-04

**Authors:** 


### Lectures on Natural and Difficult Parturition. By *Edward William Murphy*, A.M. M.D. &c. 8vo. pp. 257. London, Taylor and Walton, 1845.

Dr. Murphy is the Professor of Midwifery at University College, and his book contains thirteen of the Lectures which have been delivered to his class, and comprise by far the most important subjects which the student of Midwifery has to learn. The reason that Dr. Murphy assigns for their publication is, that "his pupils, recalling to their minds the lessons they have received, may be enabled to examine the principles and precepts delivered to them, with more care and attention than could be done from the evanescent impressions of an oral lecture." We think that any one who has the guidance of a number of young men in one branch of their studies, has a strong and legitimate inducement to publish what he teaches, for the very reason that has suggested the present book. The design which our author has had in view is well accomplished, and we feel persuaded that his pupils may refer to these Lectures as containing the substantial groundwork of the theory and practice of midwifery. Dr. Murphy calls them Επεα πτερόεντα—which is the only fugitive part about them. They are really characterised by a profound knowledge of the subjects on which they treat, and attest the careful, thoughtful, modest, and discriminating mind of their author. We hope sincerely that they will find their way beyond the precincts of Gower-street, and be perused and studied by the practitioner as well as by the student. So far as composition goes we think that, in some respects, they are better adapted for the closet than for the lecture-room. Here and there the intricate and more difficult subjects bear their character too much about them. They want breaking up—the prominent points need to be brought out and worked up to more fully—and in this way they would be simplified and catch the mind of the listening pupil.

If we are to look upon these Lectures as the literal transcripts of those which Dr. Murphy has given, we should say that his art as a lecturer was not equal to his composition as an author. We do not see the pointed, short, energetic expression which is so effective in the lectures of Sir Astley Cooper and Dr. Watson. It is, however, quite possible that Dr. Murphy is not in the habit of reading his lectures; in which case his style may vary with the circumstances which surround him, and under the immediate necessity of quick thought and spontaneous unprepared expression, it may acquire those very qualities which are so apt to be effaced in written compositions.

The Lectures are on Natural and Difficult Parturition, to which are prefixed two lectures on the Obstetric Anatomy of the Pelvis. In doing this, our author has deviated from the exact course which he pursues in the lecture-room, but he has thereby given a completeness to the subject which would have been wanting had he omitted them. A Summary of the Principles and Rules contained in the Lectures are arranged at the end of the work as Aphorisms, in which some of Denman's are included. We may notice, also, that the work is well illustrated with wood-cuts, many of which are original.

The first Lecture includes a description of the bones, articulations, and ligaments of the Pelvis. The internal surface of the ischium is described as consisting of two planes, which are separated by a line drawn from the pectineal eminence to the spine of the ischium. The anterior plane is bevelled off in a forward direction, and any body, as the head, gliding on it would mechanically be conducted forwards towards the arch of the pubes. The posterior plane has a backward curve and would tend to rotate the head backward. And thus, in natural labour, when the head enters the oblique diameter, the occiput, corresponding to the anterior plane, glides towards the arch of the pubes, whilst the sinciput moves on its plane posteriorly, the two movements co-operating simultaneously for the exit of the head.

Dr. Murphy describes the different planes of the pelvis, and notices the omission in most of the popular works of the measurements of the cavity. The planes are those of the promontory, brim, and cavity, and the effect of the differences of these planes in giving a screw-like movement to the head during labour is thus described.

"Comparing the plane of the brim with the plane of the cavity, you find the transverse measurement of the cavity diminished, while the antero-posterior and oblique distances are increased; you may also observe, that the oblique lines of the cavity approach nearer to the antero-posterior direction than those of the brim of the pelvis, so that when the head passes from the brim into the cavity, always seeking the widest space, it first rotates from the oblique of the brim into the oblique of the cavity, and as it descends, is obliged, from the convergence of the planes of the ischium still more to assume the antero-posterior direction, until, the occiput escaping under the pubic arch, it becomes fixed in this position: then the second rotation of the head from behind forwards commences, the transverse measurements of the head corresponding to the transverse of the outlet, and the longitudinal passing successively out in the antero-posterior measurement of the outlet.*" P. 14.

There is a table of measurements at the end of the first lecture in eighteen pelves not diseased, which illustrate the differences in the extent of these planes which are compatible with safe and natural parturition.

*Deviations from the Standard Pelvis.*—Our author distinguishes between deviations or irregularities, and deformities or distortions the result of disease. Amongst the deviations he describes the pelvis altogether too large, and its opposite the pelvis too small;—then again the pelvis which, being arrested in its development, retains many of the characters of the infant pelvis, and that in which the features of the male pelvis are apparent. The last of the deviations is where there is an irregularity and a want of correspondence between the different proportions of the pelvis.

In the pelvis, where every part is equally diminished, forming the too small variety, the bones of the extremities are not only short but small, and hence they are a guide to the detection of this kind of pelvis. Sometimes this small pelvis is met with in women of average height, and the author refers to three cases quoted by Dr. Rigby from Busch's Berlin Reports, in all of which labour ended fatally. There is also a case, we may add, by Dr. Ashwell, in the Guy's Hospital Reports, in which a woman with this kind of pelvis died undelivered.

The pelvis of the adult female which retains the type of the infant pelvis is illustrated by two figures, which, placed side by side, renders the resemblance obvious. The lengthened antero-posterior diameter—the shallow cavity—contracted transverse diameters both of the brim and outlet are described. There is also a variety of this Pelvis where, to the cha-

* "It is here assumed that the pubic angle is sufficiently wide to allow the occiput to pass completely under the arch, and to place the head in the antero-posterior direction."

racters of the infant pelvis—there is the evidence of a retarded growth, which contracts and lessens the whole pelvis.

Dr. Murphy attributes the acquired characteristics of the male pelvis to the active and undue exercise of the strong muscles, which causes the bones to which they are attached to be strengthened and increased. Hence, in women who have been employed from early youth in carrying heavy weights or other active muscular exertion, in accordance with the law that bone is developed to meet the strength of powerful muscles, the acetabula would have a tendency to be drawn closer to the centre, the planes of the ischia would converge more, by the bone in its growth adapting itself to the diminished distance, the ilia would be more upright, in a word, the female pelvis would put on the characters of the male pelvis. There is much serious hindrance caused in parturition by a pelvis of this kind. The head passes through the brim well—but in the cavity the narrowing of the symphysis pubis and the straightness of the sacrum impede its lateral rotation, while the increased roughness and thickness of the tubers and spines of the ischia offer further obstacles, and probably the head becomes arrested close to the outlet. Dr. M. thinks that a guide to the detection of this kind of pelvis is found in the bones of the extremities. "The wrist and ankles are large—the phalanges thick and short."

We admit the plausibility of Dr. Murphy's explanation of the cause of this form of pelvis—and we can bear testimony to the difficulty it offers to the passage of the child's head. We can only express our surprise that, if the former is right, if the pelvic bones are really influenced only by the law of their increase in proportion to the use and exercise of the muscles attached to them, we do not more frequently meet with this male configuration of pelvis. Taking the large number of women in the metropolis who, from their earliest years, have been accustomed to the undue exercise of the muscles attached to the pelvis, we ought to find a far greater proportion of male-formed pelves.

Dr. M. describes a pelvis amongst those where there is an irregularity or a want of correspondence between their different proportions which may advantageously be contrasted with the male Pelvis. Like the male pelvis, "it is also funnel-shaped; but the funnel is reversed. There is rather less space in the brim than in the standard pelvis, it is a little more oval, having its short axis (antero-posterior) less than 4 inches; but the cavity is wider, the planes of the ischia more apart, and the outlet much more open than the normal pelvis. It is almost doubtful whether a pelvis of this character may not be *slightly* diseased, and consequently that it was beginning to assume something of that shape, the extreme of which forms the distortion of rickets."

The three varieties of diseased pelves are the ovate, cordiform, and obliquely ovate. The former is the result of rickets, a disease of infancy; the second is the result of mollities ossium, a disease of the adult; whilst the most plausible explanation of the latter is that of Dr. Knox—that there has been an early arrest of development in one half of the pelvis, the other growing normally, so that the appearance of an obliquely ovate pelvis is like the union of the halves of an adult and infant pelvis, with ankylosis of the sacro-iliac joint in the arrested side. The difference between the ovate and condiform pelvis, is accounted for by considering the effect of the forces acting on the pelvis, under the different circumstances of an infant and an adult. In the latter, a line passing through the centre of gravity would fall within the pelvis, and thus the weight of the body above and the resistance of the thigh-bones below would drive the yielding points inwards, doubling up the bones towards the centre. In the former, a similar line would fall before the pelvis, and then the yielding sacrum would be pressed forward in front of the cavity, the acetabula pressing up behind it, and the pelvis become lengthened laterally. There are drawings illustrating these varieties of deformed pelvis.

The subjects we have already noticed are comprised in the two first Lectures, and we think Dr. Murphy has treated them extremely well. They well deserve the careful study of the gentlemen for whom the lectures are specially designed.

Dr. Murphy adopts Denman's division of labours, and the three stages of labour of the same author. Previous to the description of the mechanism of the dilatation of the os uteri, Dr. M. describes, at length, the arrangement of the fibres of the womb, both on its internal and external surface. This we think he rather confuses, and the effect ascribed to these muscles appears to us to be very theoretical.

Dr. M. recognizes two orbicular muscles arranged around the opening of the tubes, and a circular set around the body and cervix of the womb. The effect of the action of the two fundal muscles is shewn by a diagram, to direct a body towards and through the os uteri—and "when the circular fibres of the body and cervix contract their tendency is to render the uterus more cylindrical." Dr. M. does not conclude this lecture without modifying this arrangement, for in another place he seems to deny to the cervix the circular fibres which he speaks of as contracting, and we have in a note an admission of a number of longitudinal fibres, which have no place in his diagrams. "It is his wish," he says, "to confine the attention of the student to those only which are the most obvious, and which are quite adequate to explain the action of the uterus." The two fan-like muscles on the outside of the womb are described and shewn; and the great mass of muscular fibres which lie between the inner and the outer set, forming the bulk of the womb, are said to have no determinate direction, but may be supposed to give increased power to the others. We really believe that Dr. Murphy is just as likely to be wrong as right about the whole of this matter. If there are longitudinal fibres, they ought to be put into the diagram and accounted for; and we cannot consent for a moment to allow as anatomy that the most obvious fibres in a great complex system of them should stand sponsors for the rest, or theoretically be assumed as adequate to explain the action of the uterus during labour.

Dr. M. opposes Wigand's view of the order of uterine contraction, which he declares always to commence at the fundus, and is directed downwards, and not at the cervix and directed upwards.

Dr. M's view of the means by which the os is dilated is essentially mechanical. The fundal muscles, with the aid of the bag of waters, open up the passively resisting cervix. We have ourselves always recognized another element, which we can only call a vital act, a sort of consenting or vital dilatation. It is demonstrable sometimes when in a deformed pelvis, with a narrow brim, the bag of membranes or presenting part has never had a bearing on the cervix, and yet, as labour goes on, this part has acquired a perfect dilatability—it is felt to be unresisting, and yields at once when the head is drawn down.

Dr. M. describes the different kinds of rigid os uteri. Sometimes the structure is *tough*, and yields reluctantly. Sometimes thick cartilaginous, like Gimbernat's ligament, and it might almost be called "*the undilatable os uteri.*" Sometimes an os uteri *acquires rigidity*, having been at first dilatable. This is the result of inflammation—it becomes tender, swollen, rigid.

Our author describes the mechanism of head and face presentations in the same way as Naegele, whose opinions he has taken some pains to verify. He confirms Naegele's views of the change of the third position of the head, where the larger fontanelle is forward and to the left, (left fronto-cotyloid,) into the second position—the occiput making a spiral curve from right to left in its passage through the pelvis. At the Dublin Lying-in Hospital Dr. Murphy found that, "in nearly an equal number of cases, the head entered the brim in the third position as in the second—that, of those which descended in the third, the majority passed without any difficulty into the second, and were so expelled, while a very few remained in their original position." There is a tabular view of 74 cases in which this fact is shewn. Dr. Murphy adds his authority to that of all practical writers, that face-presentations may safely be left to Nature; and he tells us that, of the number of cases which he saw during a three years' residence at the Dublin Lying-in Hospital, "there was not a single face-position which required aid in the delivery, nor did the labour in any occupy twenty-four hours. The only danger, therefore, which might arise from these positions is the danger of meddling too much with them."

The manner in which the third stage of labour is completed, in the separation of the placenta, is said by Dr. M. to depend much upon the state of the abdominal muscles. If these are firm enough to support the fundus well, the placenta is frequently passed into the vagina by the same pain which expels the limb of the child. Dr. M. insists on the auxiliary force of the abdominal muscles and diaphragm as an important, if not an indispensable agent, in effecting this purpose; and the retention of the placenta, which is usually ascribed to uterine inertia, he thinks, is generally caused by feebleness in these structures. We do not quite concur in these views. The uterus, we fully believe, is quite competent to the unfolding and casting off of the placenta—whether its fundus be well or feebly supported by the abdominal walls. Indeed, we have witnessed it in women with large lax pendulous bellies—far too often to doubt its power in this respect. Dr. M., too, solves the riddle why after-pains occur mostly in women who have had several children, by supposing that, in these, the tonicity of the abdominal muscles is lessened or even destroyed—that the womb yields, and coagula are formed within its cavity—in expelling which, the uterus contracts and brings on after-pains. He does not ascribe this insufficient contraction primarily to the womb itself, which however we think is likely to be weakened by the distension it suffers during repeated pregnancies.

The Lectures on the Management of Labour contain those judicious regulations for the conduct of the practitioner which have been authorised by most writers on midwifery.

The seventh Lecture commences the subject of Difficult Labours. The simple duration of a labour is only one out of several circumstances which may cause it to be difficult.

"The causes which render labours difficult vary exceedingly. The delay arises sometimes in the first, sometimes in the second stage. In one instance, the constitution of the patient, or the rigidity of the passages, may retard the delivery; in another, the disproportion between the head of the child and the pelvis may impede the progress of labour. It is necessary, therefore, to classify these causes, and for this purpose we would include, under the head of '*difficult labours*,' two subdivisions—1st, That in which labour is merely prolonged beyond the average period, without being, at any time, unusually severe,—it is then called '*tedious labour*,'—2ndly, That in which, without reference to time, there is a powerful struggle carried on by the uterus to overcome some unusual resistance. This may be called by the expressive term '*laborious labour*.' The causes which produce the former are most frequently met with in the first stage of labour; those that give rise to the latter, generally occur in the second. These divisions embrace a great variety of causes, which may be classed under several heads. Tedious labour may depend either upon inefficient action of the uterus, or rigidity of the passages: and as their consideration will form the subject of the present lecture, we place these causes before you in a tabular form, in the order in which we shall consider them.

| *Inefficient action of the uterus from—* | *Rigidity of the passages—* |
|---|---|
| 1. Over-distension of the uterus. | 1. Rigid os and cervix uteri. |
| 2. Extreme obliquity of the uterus. | 2. Contracted vagina. |
| 3. Gradual escape of the liquor amnii. | 3. Rigid perinæum. |
| 4. Hysterical excitement. | |
| 5. Mental despondency. | |

"Such are the usual causes of tediousness in labour. We might add others which are less frequently met with; but it is unnecessary to propose to you a complicated classification." P. 105.

The influence of Hysterical Excitement and Mental Despondency in rendering labour tedious is well described. Perhaps, to his graphic picture of high excitement, followed by the depression of anxiety, he might have added that fainty state which so often embarrasses the first stage of labour in hysterical women, and alarms the immediate attendants.

The medical treatment is chiefly to be directed to the state of the bowels, which ought to be looked to before labour commences. During labour, however, the removal of scybalæ by an assafœtida enema is recommended. "When the bowels are unloaded, narcotics, combined with the diffusible stimulants, will more efficiently control the irritability of the patient." We think our author ought to be more explicit in defining the time and circumstance which demand "a full anodyne." We believe there is much harm arises from the exhibition of opiates in these cases, and that, instead of the "some hours refreshing sleep" being procured by it, there is very often increased restlessness, heat of skin, headache, dry brown tongue, and a further derangement of the first stage of labour. Occasionally, however, but only occasionally, it may be had recourse to with manifest advantage; but in by far the majority of cases, where the hysterical state exists, the attention to the bowels, more especially the removal of the flatus, which collects in the colon, with a diligent regard to ventilation, diet, and tranquillity, are the best adjuvants to be adopted. The tact and skill of the medical attendant in inspiring his patient with confidence, sometimes diverting her mind, and at others sustaining it by a kind and patient encouragement, are never more needed or tried than in cases of this description.

The effect of mental depression in retarding labour has often been noticed; but there is much precision given to a class of cases by our author in this paragraph on Mental Despondency. Extreme cases of the kind are rare; but the *unaccountable sudden deaths* which sometimes follow labours not marked by difficulty are to be explained in this way. Three fatal cases are related by Dr. Murphy, in which death was produced by extreme nervous shock. The deep depression in these cases, putting on the appearance to a casual observer of resignation and fortitude, is in contrast with the noisy, complaining, restless character of the hysterical patients. It is the calm of indifference and insensibility, and denotes a most dangerous state. The absence of complaint and of any distinctly bad symptom may lull the practitioner into a false security, but when once perceived, these cases need the most anxious care.

"Stimulants may be given moderately, carefully observing their effect, the temperature of the surface and extremities attended to, and the bowels (which are always constipated) relieved by warm and stimulating enemata. Ergot of rye, in moderate doses, to excite the specific action of the uterus, is useful.

"You may thus succeed in securing a favourable dilatation of the uterus, before the patient becomes exhausted; so that, in the second stage, the uterus may retain sufficient power to complete the delivery in a short time; but if this be not the case, artificial assistance becomes necessary, in order to abbreviate its duration as much as possible. When the child is partly born, you must be careful, also, not to withdraw it too rapidly from the vagina and uterus, because the danger that attends the case is not confined to the effect of tediousness in labour; you have still to guard against the syncope that may follow the complete contraction of the uterus. It should, therefore, be permitted to expel the remainder of the child very gradually, while an equable pressure is maintained by a broad bandage over the abdomen." P. 114.

The causes of delay in the second stage may arise from the head of the child, which may be placed irregularly, or be too large, or too much ossified, or hydrocephalic. They may arise too from certain states of pelvis—the masculine or diseased pelvis. These conditions are well described by our author, and also the differences which are to be observed between an arrested and an impacted head. We transcribe the paragraph which alludes to the latter.

"When the head is *arrested* in the pelvic cavity, it may be readily distinguished from the *impacted* head. In the former case, if the head be slightly pushed back, the finger can be passed with facility between the head and the pelvis, the ear may be touched, the parietal bones do not overlap each other strongly, the scalp is only puckered, or, if a tumour be formed on the presenting part, it is diffused, increases slowly, and seldom attains any magnitude. In the latter instance, when the head is *impacted*, it cannot be so easily displaced; it is impossible, without force, to pass the finger between it and the symphysis pubis; the ear cannot be felt, and the urethra is compressed. The parietal bones are strongly overlapped, and cause the sensation to the finger that is expressed by the homely simile of 'the sow's back;' a tumour grows very rapidly, and to a great size, often completely obscuring the character of the presentation; the vagina is also swollen and congested. If, however, the death of the child take place, it becomes less in size, softer, crepitant, and œdematous, while the serrated edge of the suture may be felt still more distinctly." P. 134.

Inflammation and exhaustion, the injury to the soft parts which may result from the former, and the immediate dangers which attend the latter, are the unfavourable results of laborious labours. Dr. M. describes the symptoms which indicate these states, and the general treatment to be adopted in them. When inflammation comes on, the head being in the cavity, active depletory measures, with tartar-emetic, are to be promptly used, if the patient be plethoric. If she be of weak habit, local depletion, fomentations and enemata may be had recourse to. A *moderate* opiate may advantageously be exhibited after the bowels have been relieved. When the womb is becoming exhausted, delivery must be accomplished. But much may be done to prevent the coming on of exhaustion. A moderate dose of opium, when the pains are weak, and recur at long intervals, will procure rest, and lull the over-active state of the nervous system. After a temporary rest, if the womb does not spontaneously recover, but continues to act feebly, a dose of ergot may be given to stimulate it. Dr. Murphy fences the use of this obstetric instrument with a judicious number of precautions, and cites Dr. Beatty's and Dr. Hardy's views of its fatal influence on the life of the child. This general description of the treatment of inflammation and exhaustion precedes the description of the management of those protracted labours where neither of these states have supervened, and instrumental aid is not imperatively demanded. Our author stops to consider at length " those more doubtful cases in which there seems hardly sufficient space for the head to pass safely through the pelvis. In these cases, the practice is not so clear, nor is the evidence of the profession so unanimous upon them." The discrepancy of opinion is, in other words, on those cases wherein the head is not quite arrested, " but advances so extremely slowly that it seems to be arrested." Drs. Burns, Hamilton and Campbell are cited as advocating artificial delivery by the forceps, their dread arising from too long delay, whilst William Hunter, Osborne, Denman and Collins, trust to the powers of Nature, as long as the head, under the influence of the pains, advances *ever so slowly*. Our author labours to establish the propriety of the latter practice, by a reference to facts rather than opinions. For this purpose, he derives from a statistical record of the results of British, French and German practice, that the mortality of the child, in forceps cases, is 1 in 4; whilst in Dr. Collins' able report of cases which lasted beyond twenty-four hours, the effect of leaving them to Nature was a mortality of 1 child in 5. Dr. Murphy's own experience and conclusion is thus told.

" In 5699 cases, 218 were protracted to this degree; and of these 175 were delivered naturally, and forty-one children not putrid were still-born, being one in four, nearly. Thus, then, you perceive that, taking the widest, and, we would say, the fairest view of this question, the proportion of still-born children in these difficult and protracted cases is nearly the same, whether the forceps be employed or otherwise; that the difference, if any exist, is in favour of Dr. Collins's practice of leaving these cases to nature." P. 147.

Dr. Murphy too, by a similar reference to statistics, arrives at the conclusion, that the " mortality to the mother is not increased by leaving these cases to Nature ;" and he further adds, that the post partum accidents, as sloughing of the soft parts, and the formation of fistulous communications, are due most frequently to forceps operations. With very occasional exceptions, then, Dr. Murphy adopts Dr. Collins' views, and is an advocate for non-interference in these protracted cases.

In the management of cases where the head is impacted in the cavity of the pelvis, our author cannot make the same reference to statistics, to work out from them a conclusive opinion on the merits of the forceps or perforator. One or other of these means must be employed; but " we must only hope," says Dr. M. " to determine the rule of practice by a fair examination of the question itself, by collecting the general experience of the profession, and by submitting to you the ground which would govern us as to the course to pursue." We confess our own decided preference to this form of arriving at a practical conclusion. We distrust midwifery statistics, and we feel persuaded, that it is most unsafe to yield one's judgment to them in the naked way in which they are presented to us, unless the cases of which they are composed are most diligently sifted, and their particulars accurately made out. They contain, and frequently conceal, several sources of error, which, when enclosed in the statistical columns, wear a most delusive guise, and are apt to mislead the person who depends upon them. Dr. Murphy approves of Baudelocque's experiments on the heads of still-born children, to shew that the forceps has little power in compressing and diminishing the size of the fœtal head, and agrees in regarding the instrument as an extractor. Its power in reducing the impacted head, to allow it to be drawn through the pelvis, he looks upon as very doubtful. The child's death from the compression of the head, when impacted, is very frequent, while the soft structures of the mother are exposed to the frightful dangers of contusion, sloughing, and the formation of fistulous openings. The attempt to prevent the former, increases the dangers of the latter, and thus the use of the forceps in these cases, has by most well-informed practical men been looked upon as needing the most practised discretion. The practice rests a good deal upon whether the child is alive or dead. Formerly, practitioners waited until undeniable signs of the death of the child shewed themselves, such as marked commencing putrefaction, and hence delay with its fatal results so frequently accrued. But now, by means of the stethoscope, which ought always to be used in these cases, the fœtal heart may first be heard beating, then becoming feeble and irregular, then ceasing, and the death of the child be early and certainly predicted. Now, according to Dr. Collins, if protracted labour has been properly managed, the child's death is thus announced before the symptoms in the mother become so alarming as to cause any experienced physician to lessen the head. And thus the scruples of the practitioner are allayed, and the safer practice indicated of employing the perforator rather than the forceps. Our author thus sums up his opinions on this subject.

" I trust sufficient has been placed before you to authorize the conclusions at which I have arrived, and which are now submitted to you—viz., that when the head is impacted in the pelvic cavity, it cannot be delivered by the forceps without such injury to the passages as might endanger the mother's life; that the probability of preserving the child's life is not sufficiently certain to justify an attempt which might be so hazardous; that, in the great majority of these cases, the death of the child takes place naturally, and it may be removed before symptoms dangerous to the mother present themselves; and lastly, that if it should happen that the reverse occurs, and danger to the mother—whether from exhaustion or extending inflammation—is indicated before the death of the child, that then perforation is called for, rather than render the risk to the mother a certainty, by the dangers that result from a forcible extraction by the forceps." P. 162.

The concluding Lectures are on Operative Midwifery. Dr. Murphy describes at length the use of the vectis, and quotes Mr. Gaitskill's rules for applying it. He thinks that its power as an instrument for extraction is limited, and that it is liable to some disadvantages from which the forceps is free.

There is a series of diagrams, quite original, we believe, which illustrate very well the mode of applying and using both the Vectis and Forceps. They exhibit the patient in the ordinary obstetric position on the left side.

There is a table of measurements of the forceps, which have been chiefly used in British practice, and a description with a drawing of the principal forms, beginning with Chamberlen's. The different varieties of perforators are also figured.

